# *Tetrapisispora phaffii *killer toxin is a highly specific β-glucanase that disrupts the integrity of the yeast cell wall

**DOI:** 10.1186/1475-2859-8-55

**Published:** 2009-10-27

**Authors:** Francesca Comitini, Ilaria Mannazzu, Maurizio Ciani

**Affiliations:** 1 Dipartimento SAIFET, Sez Microbiologia Alimentare, Industriale e Ambientale, Università Politecnica delle Marche, Via Brecce Bianche, 60131 Ancona, Italy; 2 Current address: Dipartimento di Scienze Ambientali Agrarie e Biotecnologie Agro-Alimentari, Università degli Studi di Sassari; Via de Nicola n 2, 07100 Sassari, Italy

## Abstract

**Background:**

Killer yeasts have been used to combat contaminating wild yeasts in food, to control pathogenic fungi in plants, and in the medical field, to develop novel antimycotics for the treatment of human and animal fungal infections. Among these killer yeasts, *Tetrapisispora phaffii *(formerly known as *Kluyveromyces phaffii*) secretes a glycoprotein known as Kpkt that is lethal to spoilage yeasts under winemaking conditions. In the present study, the mode of action of Kpkt, and the specific damage produced by this toxin on sensitive yeasts is investigated.

**Results:**

The use of castanospermine, a β-glucanase inhibitor, demonstrated that β-glucanase activity is essential for the Kpkt killer activity *in vivo*. Accordingly, Kpkt has no killer activity on either sensitive yeast spheroplasts or whole sensitive cells in the presence of isosmothic medium (0.8 molar sorbitol). Kpkt induces ultrastructural modifications in the cell wall of sensitive strains, as shown by confocal microscopy, laser-scanning electron microscopy, and atomic force microscopy. The Kpkt killer action is mediated by the glucidic portion of the toxin. This, in turn, appears to be involved both in the stronger cytocidal activity and in the selectivity for the sensitive strain shown by Kpkt compared to laminarinase.

**Conclusion:**

Collectively, these data indicate that the mode of action of Kpkt is directed towards the disruption of cell-wall integrity, and that this is mediated by a highly specific β-glucanase activity. In this, Kpkt differs from other microbial β-glucanases that do not show killer activities.

## Background

Investigations into the killer phenomenon in yeast have resulted in substantial progress towards elucidation of the intricacies of this phenomenon. In addition, they have provided valuable insights into a number of fundamental aspects of eukaryotic cell biology and virus-host-cell interactions [1-3].

Killer toxins act on sensitive cells through various mechanisms, such as inhibition of DNA replication [1], induction of membrane permeability changes [4], and arrest of the cell cycle in G1 phase. Moreover, in some cases, a toxin can interfere with cell-wall synthesis by inhibiting β-1,3-glucan synthase [5] or by hydrolyzing the major cell-wall components, β-1,3 glucans and 1,6 glucans [6-8].

To date, it is known that under competitive conditions, the killer phenomenon offers a considerable advantage to these yeast strains against other sensitive microbial cells in their ecological niches. This advantage has a basic and applied significance, and killer yeasts and their toxins have found several applications. Indeed, killer yeasts have been used to combat contaminating wild yeasts in food, and to control pathogenic fungi in plants [9-11]. In the medical field, these yeasts have been used in the development of novel antimycotics for the treatment of human and animal fungal infections [1,12,13] and in the biotyping of pathogenic yeasts and yeast-like fungi [14-16]. Moreover, killer yeasts have been used to control contaminating wild-type yeasts in the winemaking and fermentation industries. In particular, the *Kluyveromyces phaffii *(recently reclassified as *Tetrapisispora phaffii*) [17] killer toxin, known as Kpkt and known to be active in the wine-making environment, has shown a wide cytocidal spectrum towards apiculate and other spoilage yeasts [18].

Kpkt is a glycoprotein with a molecular mass of 33 kDa. Its NH_2_-terminal region shows 93% identity to β-1,3-glucanase of *S. cerevisiae*, and 80% identity to β-1,3-glucan transferase of *Candida albicans *[6]. These two proteins are involved in connecting newly synthesized β-1,3-glucan chains to existing chains, and in linking them through the β-1,6-linkage [1,19,20]. Moreover, Kpkt shows β-glucanase activity *in vitro *[6], like the NCYC 434 killer toxin of *Pichia anomala *[7,21].

In the present study, to gain further information into the mechanism of action of Kpkt, we have evaluated the effects of this toxin on the ultrastructure of the cell wall of a sensitive target, and the relationship between β-glucanase and killer activities *in vivo*.

## Methods

### Yeast strains and media

The DBVPG 6706 *Tetrapisispora phaffii *and NCYC 232 *S. cerevisiae *(DBVPG 6497) used as yeast killer strains were obtained from the Industrial Yeast Collection of the University of Perugia (DBVPG). The DBVPG 6500 *S. cerevisiae *strain was used as the sensitive strain, and the BC commercial dried yeast strain (Lallemand Inc.) was used as the non-sensitive strain. All yeast cultures were grown in YPD medium containing 20 g l^-1 ^glucose, 20 g l^-1 ^peptone and 10 g l^-1 ^yeast extract. The medium for the killer activity assay was as follows: 45 g l^-1 ^malt agar (Difco) and 0.15 mg l^-1 ^methylene blue, adjusted to pH 4.6 with 0.1 molar citrate phosphate buffer.

### Enzymatic activity inhibition in the presence of castanospermine

Kpkt was purified as already described [6] and to determine whether its activity was sensitive to the β-glucanase inhibitor castanospermine, 25 μg of Kpkt in 0.5 ml citrate phosphate buffer (pH 4.5) was mixed with 0.5 ml 25 μM castanospermine (Sigma-Aldrich, Milan) and incubated at 25°C for 1 h under static conditions. β-glucanase activity was measured, as described previously [6]. The killer activity was determined using a well-test assay [6] and viable plate counts. Briefly: DBVPG 6500 sensitive strain (10^5 ^cells ml^-1^) growing in exponential phase was added to the mixture of Kpkt and castanospermine and incubated at 25°C for 24 h. After this, the cells were harvested by centrifugation (400 × *g *for 1 min at 4°C), washed twice with sterile water, and plated on YPD.

### Kpkt effect on spheroplasts and whole cells in presence of isosmothic medium

The Kpkt killer activity was determined on sensitive yeasts, to verify the involvement of receptor sites in the killer activity. 2 × 10^6 ^cells ml^-1 ^sensitive yeast spheroplasts prepared as described by Komiyama et al. [22] were incubated at each time (24 and 36 h) at 25°C, without and with 25 μg of purified Kpkt in the YPD buffered medium, supplemented with 0.8 molar sorbitol, as osmotic stabilizer. The spheroplasts were washed three times in 0.8 molar sorbitol buffer and then used for the experimental trials. Spheroplasts were stained with 0.5% methylene blue dissolved in 50 mM KH_2_PO_4_-Na_2_HPO_4 _(pH 4.5) containing 0.8 molar sorbitol and their death was monitored by light microscopy using a Thoma-Zeiss counting chamber. At the time defined for the spheroplasts counting, aliquots of spheroplast suspensions were subjected to flow-cytometry analyses as described by Comitini et al. [6] to establish the ratio between live and dead cells following the Kpkt treatments. The positive control was obtained adding an equal amount of Kpkt, inactivated by boiling for 15 minutes, to sensitive spheroplasts. Kpkt activity was also assayed on whole sensitive cells at 25°C with 25 μg of purified toxin in 0.1 molar of citrate-phosphate buffer (pH 4.5) in presence or absence of 0.8 molar sorbitol, as osmotic stabilizer. The mortality was evaluated using viable plate counts and methylene blue stained cells in a Thoma-Zeiss counting chamber after 48 h of incubation.

### Cell-wall damage

Sensitive yeast growing exponentially at 25°C for 18 h (10^5 ^cell ml^-1^) were mixed with 46 aU of Kpkt (0.1 mg ml^-1^) in citrate phosphate buffer, pH 4.6 (Ø halo 11.0 mm), and incubated for 24 h at 25°C. To compare Kpkt with another well known killer toxin, the *S. cerevisiae *K1 toxin was used after a partial purification carried out as follows. K1 killer strain was 48-h cultured and supernatant was filter-sterilized and then 30-fold concentrated with AmiconYM10 (10-kDa cut-off membrane; Pharmacia, Uppsala, Sweden). Concentrated K1 toxin was dialysed with 10 mM citrate phosphate buffer, pH 4.6 using dialysis membrane (12 kDa, Medicell, Int. Ltd, London). The killer activity of partially purified K1 was evaluated by well test carried out at 25°C in malt agar medium buffered at pH 4.6 with 0.1 molar citrate-phosphate buffer and inoculated with the sensitive strain DBVPG 6500. In these conditions K1 produced a diameter of halo of 11 mm, comparable to that of the purified Kpkt (46 aU).

Different cell-wall damage with the sensitive yeast was obtained by boiling cells for 10 min, or treating them with 70% ethanol or 2 mg ml^-1 ^Zymolyase^® ^(ICN, Biomedicals Inc., UK). After these incubations, the cells were collected by centrifugation, washed two times in sterile water, re-suspended in 30 μl 2 μM solution of Calcofluor White^® ^Stain (Sigma-Aldrich, Milan) and observed under a fluorescence microscope (Biorad, MRC 1024 UV) provided with an ebq 100 isolated lamp, a UV filter, and 25× and 100× objectives.

The potential damage to the envelope of sensitive cells resulting from Kpkt treatment was also investigated by scanning electron microscopy (SEM). Initially, sensitive cells grown overnight were counted using a Thoma-Zeiss chamber, and 10^5 ^cell ml^-1 ^were incubated at 25°C for 24 h without and with purified Kpkt (Ø halo 11.0 mm). After incubation, the cells were harvested by centrifugation, washed in sterile water and progressively dehydrated by successive soaking in 10%, 30%, 50%, 70%, 80%, 90%, 95% and 100% ethanol. Soaking in isopentyl acetate was performed before they were critical point dried in CO_2 _using a Polaron CPD 7501. For each sample, the filters were then attached to the large scanning microscopy stubs and coated with gold-palladium by cathode spreading in a Polaron Sputter Coater. Sample observation was performed using a Philips XL20 SEM operating at a voltage of 10 kV to 20 kV.

To confirm the damage produced by Kpkt on the cell wall of sensitive cells, and to examine it in more detail, the samples previously observed by SEM were used for an atomic force microscopy (AFM) study. Briefly, 10 μl of 2 × 10^6 ^cells were collected from each sample, applied to a Corning microscope slide, and observed by AFM. The images were analysed using the BG-one software.

### β-glucanase and Kpkt enzymatic activity

The effects of increasing concentrations of laminarinase were studied in both the sensitive and the non-sensitive yeast strains, by evaluation of the threshold values at which the hydrolytic enzyme is lethal for yeast cells. Laminarinase (Sigma-Aldrich) was used at concentrations of 0.05, 2.5, 12.5, and 25 mg ml^-1 ^(0.25, 12.5, 62.5, 125 IU) in a final volume of 1 ml in 50 mM sodium acetate buffer (pH 4.5). Suspensions of both sensitive and non-sensitive cells (10^6 ^cell ml^-1^) were added to each hydrolytic enzyme concentration and incubated for 24 h at 37°C. After this time, the viability of the yeast cells was analysed by viable plate counting and by flow cytometry upon propidium iodide staining. A positive control was added with yeast cells not treated with laminarinase.

### Effects of endoglycosidase-H on Kpkt killer activity

KpKt was treated with endoglycosidase H (45 IU [mg protein]^-1^; ICN Biomedicals) and the killer activity of the residual protein portion of Kpkt was determined. The assays were performed following the procedure described by Elgersma et al. [23], modified as follows: 2 μl endoglycosidase-H (0.01 IU μl^-1^) was added to 25 μg of Kpkt and 73 μl buffer (150 mM sodium citrate, 1 mM PMSF, 10 μM pepstatin, 5 mM sodium azide, pH 5.9). A positive control with heat-inactivated Kpkt was prepared. A negative control was prepared by mixing 25 μg of Kpkt with 75 μl buffer in the absence of the lytic enzyme.

All of the samples were incubated at 28°C for 20 h and 48 h. After this, the mixtures were added to the sensitive cells (2 × 10^5 ^cells ml^-1^) and than subjected to viable plate counts and the well test to assess killer activity.

## Results

### Castanospermine inhibits Kpkt killer action

While the β-glucanase activity of Kpkt has already been demonstrated *in vitro *[6], its involvement in Kpkt killer action was here assessed *in vivo *in the presence of castanospermine, a specific β-glucanase inhibitor. As shown in Table 1, at 25 μM castanospermine, there was a dramatic reduction in the glucanase activity, which was associated with a complete loss of Kpkt killer activity, confirming a direct relationship between its β-glucanase and killer activities.

**Table 1:** Effects of the β-glucanase inhibitor castanospermine on Kpkt killer activity.

Purified Kpkt	Enzymatic activity (g l^-1 ^glucose)	Killer activity Ø halo in well test (mm)	Viable cell counts CFU ml^-1^ (sensitive cells)
No castanospermine	0.032	11.0	0.5 × 10^2^
25 μM castanospermine	0.0001	0.0	1.5 × 10^5^

The Kpkt treatment was carried out with 25 μg toxin for 24 h at 25°C. The initial inoculum of the sensitive strain was 10^5 ^cell ml^-1^. Growth of the sensitive cells was measured through viable plate counts.

### The cell wall is the target of Kpkt

**Figure 1:**
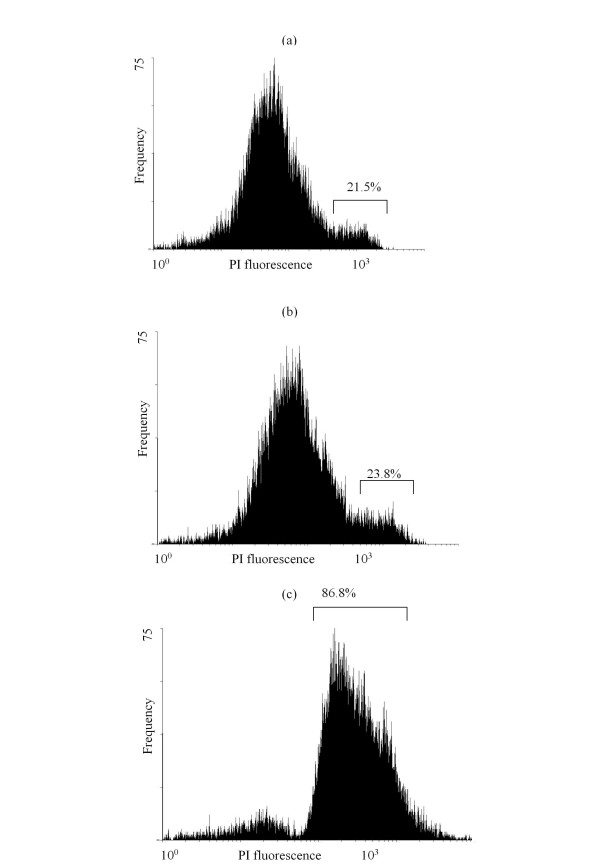
**Flow cytometry analysis of the sensitive strain**. 10^6 ^cells ml^-1 ^treated with 25 μg Kpkt for 24 h at 20°C, showing propidium iodide stained DBVPG 6500: (a) spheroplasts with heat-inactivated Kpkt; (b) spheroplast with Kpkt; and (c) sensitive whole cells with Kpkt. The peak on the left on the x axis is made up of viable spheroplasts/cell (not permeable to propidium iodide). The peak on the right of the x axis is made up of dead spheroplasts/cells (permeable to propidium iodide). Percentages represent the mortality after flow-cytometry analyses.

To further elucidate the mechanism of action of Kpkt, its killer activity was investigated with spheroplasts of sensitive cells. Figure 1 shows the results of flow cytometry of propidium-iodide-stained spheroplasts after Kpkt treatment. It is known that propidium iodide stains the nucleic acids of dead or damaged cells, providing an indirect measure of cell membrane integrity [24]. Spheroplasts of the sensitive strain treated with inactivated and active Kpkt (Figures 1a, b, respectively) showed the same profiles of propidium iodide fluorescence, indicating that in contrast to whole sensitive cells, spheroplasts are resistant to this toxin (Figure 1c). These results were confirmed with viable plate counts (Table 2). The evaluation of Kpkt activity on whole sensitive cells in the presence of 0.8 molar sorbitol as an osmotic stabilizer, confirmed that its zymocidal action is directed towards the cell wall (Table 3). Indeed, whole sensitive cells treated with Kpkt survived in an isosmothic environment, while they died without an osmotic stabilizer.

**Table 2:** Effect of Kpkt on spheroplasts

Trial	% dead cells^a^
Spheroplasts + heat treated Kpkt	0.29 ± 0.23
Spheroplasts + Kpkt	1.39 ± 0.42
Whole cells + Kpkt	98.5 ± 0.77

The initial inoculum (10^6 ^cell ml^-1^) was considered as 100%. Data are means ± SD of three experiments.

**Table 3:** Kpkt activity in the presence and absence of sorbitol as an osmotic stabilizer

Medium	% Dead cells^a^	
	Thoma-Zeiss counting chamber	Viable cell counts After 48 h
Whole sensitive cells + Kpkt in 0.8 molar sorbitol	5.4 ± 1.0	4.0 ± 0.8
Whole sensitive cells + Kpkt	55.4 ± 5.2	93.9 ± 3.0

^a ^The initial inoculum (10^6 ^cell ml^-1^) was considered as 100%. Data are means ± SD of three experiments.

**Figure 2:**
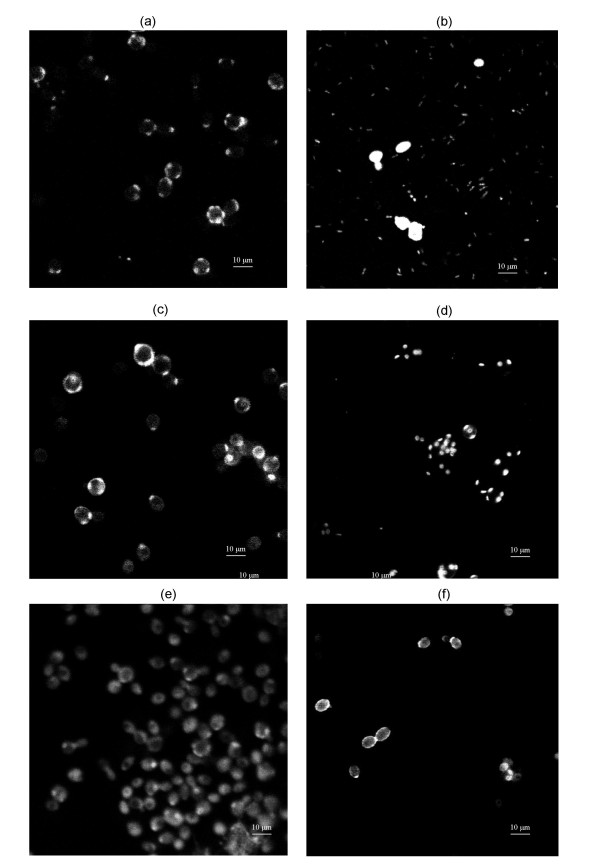
**Calcofluor White^® ^staining for cell-wall damage**. (a) untreated cells; (b) cells heat treated at 100°C for 10 min; (c) cells treated with 70% ethanol for 3 h; (d) cells broken after treatment for 2 h at 37°C with 0.02 UI of zymolyase; (e) cells treated for 24 h with 50 μg purified Kpkt; and (f) cells treated for 24 h with partially purified K1.

Calcofluor White^® ^Stain selectively links chitin in the cell wall and allows the observation of changes in envelope morphology. This was used to investigate the effects of Kpkt on the cell wall, and to provide further information on its mode of action. As shown in Figure 2, untreated and viable sensitive yeast cells showed a uniform and homogenous shape, with a continuous perimeter and fluorescence localized mainly to the bud scars (Figure 2a). Ethanol treatment damaged the cell membrane but not the cell wall, which maintained the same aspect as for untreated and viable cells, with fluorescence localized to the bud scars (Figure 2c). In contrast, the fluorescence was not well localized when the cells were killed at 100°C, indicating the complete disintegration of the cell wall (Figure 2d). Sensitive cells treated with Zymoliase^®^, a β-1,3-glucan laminarin-penta-hydrolase that lyses yeast cell walls, showed a discontinuous cell perimeter (Figure 2b). Upon Kpkt treatment, the cell wall was characterized by diffuse fluorescence, indicating the effective damage by Kpkt to the envelop of the sensitive strain (Figure 2e). However, treatment of the sensitive cells with the K1 killer toxin (Figure 2f), the mode of action of which is directed towards the cell membrane, did not show any modifications of the cell-wall structure.

### Kpkt affects cell-wall ultrastructure

**Figure 3:**
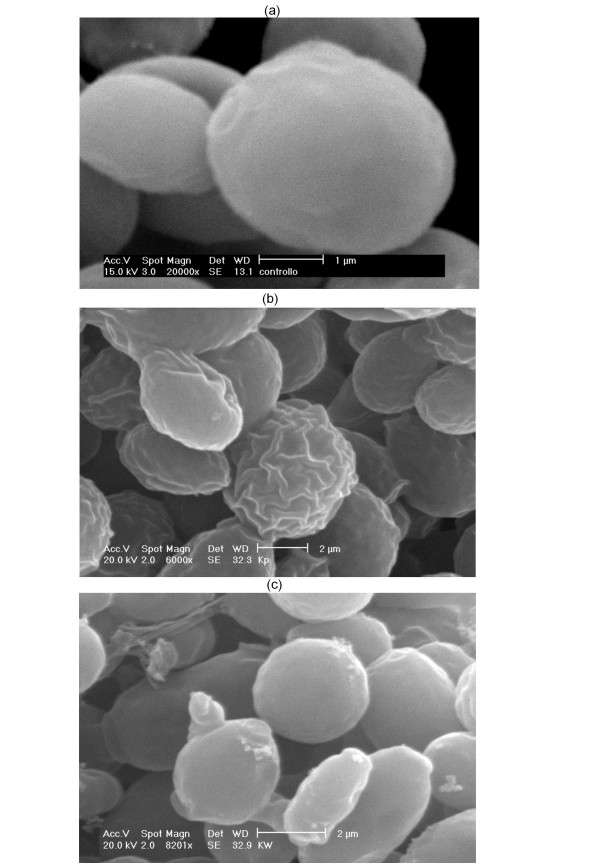
**Scanning electron microscopy analysis of the *S. cerevisiae *sensitive strain**. (a) Untreated cells; (b) cells treated for 24 h with 46 aU Kpkt; and (c) cells treated for 24 h with 46 aU K1.

The effects of Kpkt on the cell wall of sensitive cells were investigated by scanning electron microscopy (SEM) and atomic force microscopy (AFM). SEM analysis showed that after Kpkt treatment, the sensitive cells have a rough surface and an irregular and uneven shape (Figure 3). SEM specimens are coated with a film of evaporated gold that is approximately 20 nm thick, and this application might mask possible ultra-structural changes in the envelope [25,26]. Thus, to analyze the ultra-structural modifications produced by Kpkt in more detail, AFM was used.

**Figure 4:**
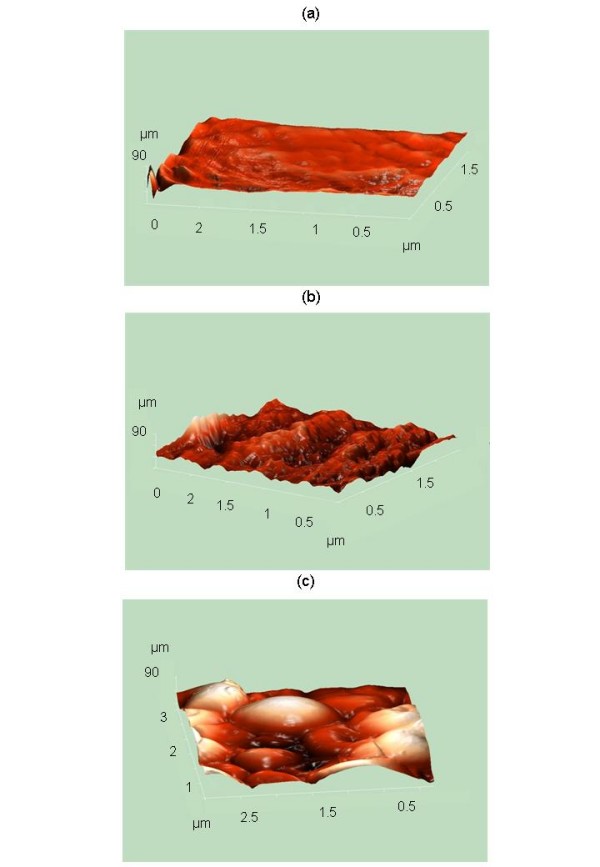
**Scanning of the surface (6.25 μm^2^) of *S. cerevisiae *cells by a semi-contact cantilever with AFM**. (a) Untreated cells; (b) cells treated for 24 h with 100 μg ml^-1 ^purified Kpkt; and (c) cells treated for 24 h with 46 aU K1.

With AFM, the cells were visualized at a very high resolution, both uncoated and alive, producing excellent images that allowed semi-quantitative studies of the roughness of the envelope of the sensitive yeasts. AFM analysis indicated that the average roughness of the untreated cells was very different to that of the samples treated with Kpkt. While the untreated cells and K1-treated cells showed similar roughness (4.39 and 4.88 nm, respectively), the cell surface of the Kpkt-treated cells appeared irregular, showing a high level of roughness (248.27 nm), with a high mean value. Moreover, the high entropy level, which quantifies the information concerning the random events in the system, highlighted a higher level of abnormality in the Kpkt-treated sensitive cells (8.146) compared with untreated cells (7.174) and K1-treated cells (6.347) (adimensional parameter). The images from AFM confirmed the results obtained by SEM (Figure 4).

### Kpkt has a highly specific activity and its glucidic fraction mediates its killer action

Table 4 shows the results of the treatments of two different yeast strains with Kpkt in comparison with increasing concentrations of a commercial laminarinase. Laminarinase killed Kpkt sensitive and resistant yeasts in a dose-dependent fashion. In contrast, Kpkt acted selectively on the sensitive strain, and as expected, it did not have any effects on the resistant yeast strain. Moreover, comparison between Kpkt and laminarinase showed that the cytocidal effect of commercial laminarinase is 500-fold lower than that of Kpkt. Thus, while Kpkt enzymatic activity *in vitro *is comparable to that of laminarinase [6], its cytocidal activity *in vivo *is significantly higher than that of laminarinase. Results of endoglycosidase-H killer toxin treatments, shown in Table 5, indicate that the removal of Kpkt glucidic residues results in a loss in its killer activity, demonstrating that the sugar fraction is involved in the mode of action of this killer toxin.

**Table 4:** Effects of increasing concentrations of laminarinase on Kpkt sensitive and resistant strains: comparison with Kpkt.

Sample	Concentration (mg ml^-1^)	% dead cells	
		**Kpkt-sensitive strain**	**Kpkt-resistant** **strain**

Kpkt	0.05	65.4	5.4

Laminarinase	0.05	1.3	3.5
	2.5	15.2	17.2
	12.5	40.8	52.7
	25	63.7	78.4

Percentages of dead cells was evaluated by flow cytometry after propidium iodide staining.

**Table 5:** Effects of endoglycosidase-H treatment on Kpkt killer activity.

Conditions	After 24 h incubation (CFU × 10^5 ^ml^-1^)	After 48 h incubation (CFU × 10^5 ^ml^-1^)
Without Kpkt	17.0 ± 1.27	56.0 ± 8.48
With Kpkt	0.5 ± 0.14	0.03 ± 0.016
With Kpkt + Endo H	6.4 ± 0.42	6.7 ± 0.56
With Kpkt and without Endo H	0.29 ± 0.098	0.43 ± 0.013

The initial inoculum of sensitive cells was 2 × 10^5 ^cell ml^-1^. Data are means ± SD of three experiments.

## Discussion

Kpkt is a glycoprotein that is secreted by *T. phaffii *and recognizes β-1,3- and β-1,6-branched glucans as its receptor site on the cell wall of the sensitive target. The NH_2_-terminal amino-acid sequence of this killer toxin shares high homology with proteins belonging to the β-glucanase lytic enzyme family, and the β-glucanase activity of Kpkt has been established *in vitro *[6].

Previous studies have reported a broad spectrum of activity for Kpkt, and demonstrated its stability under winemaking conditions, indicating its promising features for use as a novel antiseptic agent in the wine and beverage industries [10,18,27]. In the present study, the mode of action Kpkt was characterized in view of its potential application as a natural antimicrobial agent.

The inhibitory effects of castanospermine on both the enzymatic and killer activities of Kpkt confirmed that β-glucanase activity mediates Kpkt killer action *in vivo*. On this basis, we hypothesized that Kpkt has a hydrolytic activity on β-glucans within the cell wall of sensitive yeasts. The inability of Kpkt to kill spheroplasts confirmed this hypothesis and highlighted the role of the cell wall in interactions with the toxin. Moreover, this result indicated that in contrast to the *S. cerevisiae *K1 and K2 killer toxins [28], Kpkt does not have any action on the plasma membrane. The disappearance of the Kpkt killer effect on whole sensitive cells in an isotonic environment indicated that the cell wall is the only target of its mode of action.

This study of the cell envelope of Kpkt-treated sensitive cells using Calcofluor White^® ^Stain, and the SEM and AFM examinations, highlighted the specific damage produced by this toxin on the cell wall of sensitive strains. These techniques revealed that Kpkt produces a high level of roughness of the cell wall of the sensitive target, and causes changes in cell morphology. Moreover, it was seen that the cell-wall degrading activity of Kpkt is completely different to that of the *S. cerevisiae *K1 killer toxin, where the mechanism of action on the permeability of the cell membrane is known [23,29].

The strict relationship between Kpkt killer and β-glucanase activities indicates that the cell wall of the sensitive strain represents the specific binding site and the target of both killer and β-glucanase activities.

The comparison of the cytocidal activity of laminarinase and Kpkt showed for Kpkt a higher specific activity toward the yeast cell target. Indeed, a concentration of Kpkt 500-fold lower than that of laminarinase was sufficient to obtain a comparable killer action. Moreover, Kpkt showed a different selective action towards the yeast cell target that towards laminarinase.

## Conclusion

In conclusion, these data indicate that the mode of action of Kpkt is directed toward the cell wall, and that it is different from that of a commercial β-glucanase such as laminarinase. Both high specific cytocidal activity and selectivity of Kpkt differentiate the β-glucanase of *T. phaffii *from other microbial β-glucanases that do not show killer action and might have biotechnological applications [30,31]. In addition, our data indicate that the glucidic portion of Kpkt has an important role in its killer action. We therefore hypothesize that both the selectivity and specific activity of Kpkt depend on the glucidic fraction of the toxin.

## Competing interests

The authors declare that they have no competing interests.

## Authors' contributions

FC, IM and MC contributed equally to this manuscript. FC performed the experimental part of the work. FC, IM and MC carried out the analysis of the data and wrote the manuscript. All authors participated in the design and discussion of the research. MC directed the research. All the authors have read and approved the final manuscript.
